# Review of major trials of acute blood pressure management in stroke

**DOI:** 10.1177/0271678X211004310

**Published:** 2021-03-24

**Authors:** Thompson G Robinson, Jatinder S Minhas, Joseph Miller

**Affiliations:** 1 Department of Cardiovascular Sciences, University of Leicester, Leicester, UK; 2 National Institute for Health Research Leicester Biomedical Research Centre, The Glenfield Hospital, Leicester, UK; 3 Department of Emergency Medicine, Henry Ford Hospital and Wayne State University, Detroit, MI, USA

**Keywords:** Blood pressure, acute stroke, intracerebral haemorrhage, randomised controlled trials, pharmacology

## Abstract

Over the last two decades, there have been a number of major landmark clinical trials, classified as “major” as they sought to address clear clinical practice driven questions, in a pragmatic yet robust trial design, using a large powered sample size (n > 1000), in order to help improve patient outcome through informing guidelines. A commonality across all stroke sub-types included in these trials is the tendency to acute hypertensive crises within the acute stroke period. This phenomenon is associated with greater stroke complications and worsened overall prognosis. Multiple trials have attempted to address the issue of acute blood pressure management during the acute stroke period, with consideration for timing, magnitude of lowering, agent and relationship to other interventions. This review will consider the major clinical trials performed in ischaemic and haemorrhagic stroke that test the hypothesis that acute BP reduction improves clinical outcomes.

## Introduction

While long-term blood pressure (BP) control has significant effects on secondary stroke prevention, the effect of acute BP reduction on clinical outcomes has been less certain. The majority of patients with acute stroke have a hypertensive response.^1^ In haemorrhagic stroke, acute hypertension is particularly common and patients frequently present with systolic BP (SBP) greater than 180 mmHg.^1^ Furthermore, observational data reveal that the hypertensive response is associated with poorer prognosis in both ischaemic and haemorrhagic stroke.^2,3^

Whether acute BP reduction can ameliorate the neurological injury in stroke and prevent further deterioration has remained an important hypothesis, and multiple trials have attempted to answer this question. Importantly, unified outcome measures of function exist for stroke trials, including the well validated and widely used modified Rankin Scale, a quantitative measure of level of disability and dependence. Good outcome is commonly defined as scores of 0-2 and poor outcome scores of 3-6. In this section, we review the major clinical trials performed in ischaemic and haemorrhagic stroke that test the hypothesis that acute BP reduction improves clinical outcomes.

## Ischaemic stroke trials

No large, randomised trial has demonstrated clear benefit of BP reduction in the early management of ischaemic stroke. Here we present the largest clinical trials testing BP reduction in the acute management of ischaemic stroke (Table 1). The first large trial was the Scandinavian Candesartan Acute Stroke Trial [SCAST], which tested the efficacy of BP reduction within the first 24 hours since stroke symptom onset.^4^ In the SCAST trial, researchers randomised 2,029 patients with acute stroke within 30 hours of symptom onset to receive either candesartan cilexetil or placebo for 7 days. The mean time from symptom onset to randomization in the trial was 18 hours, and the mean enrolment BP was 171/90 mmHg. Most patients had ischaemic stroke (85%), but the trial did include haemorrhagic stroke patients. Mean BP (standard deviation [SD]) in the candesartan arm was 147/82 mmHg (23/14) compared to 152/84 mmHg (22/14) in the placebo arm. For the first co-primary outcome of functional status at 6-months, adjusted analysis showed a non-statistically significant shift in favour of placebo (odds ratio 1.17, 95% CI 1.00–1.38). The second co-primary outcome of composite vascular events demonstrated no significant difference between candesartan and placebo (adjusted hazards ratio 1.09, 95% CI 0.84–1.41). Subgroup analysis suggested that there was a signal of reduced composite cardiovascular events (recurrent stroke, myocardial infarction or vascular death) among patients who were treated with candesartan within 6 hours of symptom onset.

**Table 1.** Major clinical trials of blood pressure reduction in acute ischaemic stroke.

Trial	Design	Subjects	Time	Agent	Outcome	Results
Candesartan for treatment of acute stroke (SCAST)^4^	Randomised double blind	2029	<30 hours	Candesartan vs. placebo	Adverse events and functional outcome at 6 months	No difference in neurological recovery or adverse events
China Antihypertensive Trial in Acute Ischaemic Stroke (CATIS)^5^	Randomised double blind	4071	<48 hours	Tiered treatment for 10%–25% SBP reduction vs. no treatment	Death and functional outcome at 14 and 90 days	No difference in death or major disability
Efficacy of Nitric Oxide in Stroke (ENOS)^6^ Prehospital transdermal GTN in patients with ultra-acute presumed stroke (RIGHT-2)^9^ Intensive BP reduction with intravenous thrombolysis therapy for AIS (ENCHANTED)^10^	Randomised controlled Randomised controlled, blinded-endpoint Randomised controlled, blinded-endpoint	4011 (3382 ischaemic) 1149 (597 ischaemic) 2227	<48 hours <4 hours <6 hours	7 days of GTN vs. no GTN 4 days of transdermal GTN vs. sham dressing Intensive (130–140 mmHg systolic) vs. guideline (<180 mmHg) by local protocol	Functional outcome at 90 days Functional outcome at 90 days Functional outcome at 90 days	Acceptable safety but no improvement in functional outcome No improvement in functional outcome Acceptable safety but no improvement in functional outcome

GTN: transdermal glyceryl trinitrate; BP: blood pressure; AIS: acute ischaemic stroke.

A second major trial was the China Antihypertensive Trial in Acute Ischaemic Stroke (CATIS), which was a randomised controlled trial of 4071 patients with acute ischaemic stroke and SBP between 140 and 220 mmHg. The CATIS trial randomised patients into either a cohort with 10–25% BP lowering or a cohort with home and inpatient antihypertensive medications withheld.^5^ The enrolment window was up to 48 hours after symptom onset, but the mean time to enrolment was 15 ± 13 hours. In the treatment arm, antihypertensive therapy consisted of a tiered combination of medications: first-line intravenous enalaprilat, second-line calcium channel blockers, and third-line diuretics. The primary outcome was the event rate of either death or major disability, defined as a modified Rankin scale score at 14-days or hospital discharge ≥3. The mean SBP reduction in the CATIS treatment arm was 12.7% (166.7 to 144.7 mmHg) at 24 hours compared to 7.2% (165.6 to 152.9 mmHg) in the control arm. The trial failed to show a reduction in death or major disability (odds ratio 0.99, 95% CI 0.86–1.15) with antihypertensive treatment at either 14-days or hospital discharge or at 3-months. Subgroup analysis suggested reduced death and disability at 3-months in patients randomised after 24 hours (odds ratio 0.73, 95% CI 0.55–0.97). The trial did not find higher adverse events in the treatment arm.

The third large clinical trial of BP reduction in acute stroke was the Efficacy of Nitric Oxide in Stroke (ENOS).^6^ This trial enrolled 4011 patients with acute ischaemic or haemorrhagic stroke and elevated SBP (140–220 mmHg) within 48 hours of symptoms onset. The trial randomised patients to transdermal glyceryl trinitrate (GTN) for 7 days (5 mg per day) versus no GTN. A subset of patients were further randomised to continue or hold pre-existing home antihypertensive medications. Preclinical data had shown that GTN, a nitric oxide donor, modulates vascular and neuronal function in addition to its vasodilatory effects.^7^ The trial excluded patients with a definite need to start or continue antihypertensive medications and patients with pure sensory findings or isolated dysphagia. The primary outcome was the modified Rankin scale at 90 days. Median time to randomization was 26 hours, and average Scandinavian Stroke Scale was 33.7, equivalent to a National Institute of Health Stroke Scale score of 11.2 (both widely used measures of assessing severity of stroke associated neurological deficit). Mean enrolment BP was 167/90 mmHg, with a significantly greater reduction in SBP over the first day in the GTN compared to no GTN cohort (difference 7.0 mmHg, 95% CI 5.6–8.5 mmHg). For the primary outcome, functional outcome at 90-days as determined by the modified Rankin scale, there was no difference between the GTN and no-GTN cohorts (adjusted odds ratio 1.01, 95% CI 0.91–1.13). Furthermore, rates of death and secondary outcomes, such as cognition and quality of life, were no different.

The ENOS trial group additionally published a pre-planned subgroup analysis of patients within the trial that were randomised within 6 hours of stroke onset.^8^ There were 273 patients, of whom 76% has ischaemic stroke. Mean BP reduction with the first dose of GTN was 9.4/3.3 mmHg. At 90-days, patients randomised to GTN had a significant shift towards better functional outcome, as determined by the modified Rankin scale (adjusted common odds ratio 0.51, 95% CI 0.32–0.80). Adverse events were less common in the GTN group compared to those that did not receive GTN.

The fourth large clinical trial of blood pressure reduction, the only to lower in the pre-hospital hyperacute period (<4 hours), was the pre-hospital transdermal GTN in patients with ultra-acute presumed stroke (RIGHT-2) trial.^9^ The trial enrolled 1149 participants, 597 with confirmed ischaemic stroke, with a median randomization time of 71 minutes. The trial randomised patients to a GTN or sham patch for 4 days. In the GTN group, SBP was lowered by 5.8 mmHg and diastolic BP by 2.6 mmHg as compared to the sham group. Overall, GTN did not seem to improve functional outcome (adjusted odds ratio 1.25, 95% CI 0.97–1.60) in the final diagnosis of stroke or TIA group.

Lastly, the BP arm of the ENCHANTED trial assessed intensive vs. guideline BP reduction in intravenous thrombolysis eligible and treated acute ischaemic stroke patients.^10^ 2196 eligible patients were randomised to intensive (130–140mmHg) vs. guideline (<180mmHg) BP lowering using pharmacological therapy usually delivered through local protocols. Overall, although intensive BP lowering was safe, the reduced intracranial haemorrhage rates did not manifest as an improvement in clinical outcomes as compared to guideline treatment.

This clinical trial review is not exhaustive but covers the published, large clinical trials that have tested early BP reduction (<48 hours) in the management of acute ischaemic stroke. These trials have been adequately powered to test the hypothesis that BP reduction improves functional outcomes. Though heterogeneous in the antihypertensive agents used, the trials have consistently shown no sustained improvement in functional outcomes with modest BP reductions. Therefore, overall, current international guidelines only recommend acute BP reduction in ischaemic stroke for limited indications: before alteplase treatment (<185mmHg systolic and <110mmHg diastolic), when comorbid conditions exist (e.g. acute coronary event, lower BP initially by 15%) and in those ineligible for alteplase and no comorbid disease (if BP > 220/10 mmHg, lowering by 15% is acceptable).^11^ Nonetheless, key questions remain, including the potential role for very early initiation of BP reduction (<6 hours) and whether other drug classes may be efficacious. The antihypertensive medications primarily used in these trials were nitrates, angiotensin II receptor blockers and angiotensin-converting enzyme inhibitors.

## Haemorrhagic stroke trials

Given the potential to reduce hematoma expansion and further neurological injury, early intensive BP reduction has been an attractive potential therapy for haemorrhagic stroke (Table 2). Two small trials, the INTensive Blood Pressure Reduction in Acute Cerebral Haemorrhage Trial (INTERACT) study and the Antihypertensive Treatment in Acute Cerebral Haemorrhage (ATACH) study showed safety and potential efficacy with intensive BP lowering in haemorrhagic stroke.^12,13^ The INTERACT trial demonstrated reduced haematoma volume with intensive BP reduction, a finding that could translate to increased functional recovery if replicated in a large trial. Hence, both of these small trials led to large, multicentre trials over the past 5 years.

**Table 2.** Major clinical trials of blood pressure reduction in acute haemorrhagic stroke.

Trial, date	Design	Subjects	Time	Agent	Outcome	Results
Efficacy of Nitric Oxide in Stroke (ENOS)^17^	Randomised controlled	620 ICH of 4011 total	<48 hours	7 days of GTN vs. no GTN	Functional outcome at 90 days	Acceptable safety but no improvement in functional outcome
Rapid BP lowering in patients with acute ICH (INTERACT2)^14^	Randomised open-label with blinded end-point	2839	<6 hours	Open-label: target SBP 140 vs. 180 mmHg	Death or major disability at 90 days	Intensive lowering of BP did not reduce death or major disability but suggests improved functional outcomes
Antihypertensive treatment of acute ICH II (ATACH II)^15^	Randomised controlled trial	1000	<4.5 hours	Nicardipine target SBP 140 vs. 180 mmHg	Death or major disability at 90 days	Intensive lowering of BP did not reduce death or major disability

ICH: intracerebral hemorrhage; GTN: transdermal glyceryl trinitrate.

INTERACT2 enrolled 2839 patients with spontaneous intracerebral haemorrhage and acute systolic hypertension within 6 hours of symptom onset.^14^ The trial randomised patients to a target SBP <140 mmHg versus <180 mmHg. Antihypertensive treatment was open-label and at the discretion of treating physicians. Mean enrolment BP was 179/101 mmHg. Within one hour of enrolment, mean SBP was 150 mmHg in the intensive treatment arm versus 160 mmHg in the standard treatment arm. INTERACT2 showed no reduction in the primary outcome of death or severe disability, defined as a score of 3 to 6 on the modified Rankin scale (odds ratio for greater disability with intensive treatment 0.87, 95% CI 0.77–1.00). However, an ordinal analysis of the modified Rankin scale scores indicated a favorable shift with intensive BP lowering being associated with improved outcomes (pooled odds ratio for shift to higher modified Rankin score 0.87; 95% CI 0.77 to 1.00), which was not associated with early neurological deterioration or adverse events.

ATACH II tested the hypothesis that intensive BP lowering early in the care of haemorrhagic stroke would improve functional outcomes.^15^ The trial randomised 1000 patients within 4.5 hours of symptom onset to a standard treatment group (target SBP 140 - 179 mmHg) versus an intensive lowering arm (target SBP 110–139 mmHg). The trial used intravenous nicardipine as first-line antihypertensive therapy, and the primary outcome was death or disability, determined by a modified Rankin scale score of 4 to 6 at 3 months. Mean enrolment SBP was 183.5 mmHg, with average SBP levels during the first two hours of 128.9 mmHg in the intensive and 141.1 mmHg in the standard treatment arm. Due to futility, the trial stopped after a pre-specified interim analysis. The primary outcome occurred in 38.7% of patients in the intensive BP treatment arm and in 37.7% of those in the standard arm (relative risk 1.04, 95% CI 0.85 to 1.27). Adverse events during the 3 months after randomisation were higher in the intensive-treatment arm (25.6% versus 20.0%, adjusted relative risk, 1.30, 95% CI 1.0–1.7), largely involving renal adverse events associated with a rise in serum creatinine.^15^

A recent preplanned pooled analysis of the individual patient-level data from the main phase of the INTERACT2 and the ATACH II trials assessed the independent associations between three post-randomization SBP summary measures.^16^ These included: magnitude of reduction in 1 hour, mean achieved SBP and variability in SBP between 1 hour and 24 hours and their respective associations with the primary outcome of functional status at 90 days. This pooled analysis showed that achieved SBP was continuously associated with functional status (improvement per 10 mmHg increased OR 0.90 (95% CI 0.87–0.94)) and achieving early and stable SBP was safe and associated with favourable outcomes in haemorrhagic stroke patients. These data provided from the INTERACT2 and ATACH II trials has generated debate as to the balance of benefits and risks of intensive BP lowering in acute ICH. The pooled trial provides greater granularity as to a subset for whom intervention may be beneficial – thus paving the way to identify sub-groups of mild-moderate ICH who may benefit.

The ENOS investigators additionally published a pre-planned subgroup analysis of patients included in the trial with haemorrhagic stroke.^17^ There were 629 haemorrhagic stroke patients that were randomised to GTN versus no GTN. The mean time to randomisation was 15 hours and the mean enrolling BP was 172/93 mmHg. BP reduction over the first 24 hours was greater in the GTN cohort (difference −7.5/−4.2 mmHg). Overall, there was no difference in functional improvement with GTN (adjusted odds ratio for worse outcome with GTN 1.04, 95% CI 0.78–1.37). As with the overall trial, subgroup analysis of the 61 patients randomised within 6 hours suggested a positive effect of GTN on functional recovery (odds ratio 0.22, 95% CI 0.07–0.69). More recently, pre-specified subgroup analysis from the RIGHT-2 trial demonstrated in those with ICH (145 of the 1149 participants in the RIGHT-2 trial), that GTN worsened outcomes (Mann-Whitney difference 0.18, 95% CI 0.01–0.35).^18^

Current guidelines advocate lowering of SBP in ICH patients to 140 mmHg with suggestions it can be effective for improving functional outcome.^19^ Both INTERACT2 and ATACH II had a large proportion of small volume haemorrhages (median volume 11 and 10 mL respectively), raising the question of generalizability to more severe ICH patients with large hematomas. Although ENOS had a signal of efficacy with treatment within 6 hours, the larger trials enrolled all patients in this time window and did not demonstrate efficacy. Although one could hypothesise that nitric oxide donors or other specific antihypertensive agents may have efficacy, the potential for efficacy with intensive BP lowering alone is minimal. Ultra-acute delivery of vasoactive lowering regimens has demonstrated signal for harm in ICH and further prospective randomised data is required. Ultimately, neuroprotective strategies targeting the ultra-acute and acute periods post ICH are needed to minimise the significant morbidity and mortality associated with this stroke sub-type.

## Relevance of cerebral autoregulation status in AIS and ICH

Beyond major trial data, targeted mechanistic cerebral blood flow based studies have provided insight into the importance of cerebral autoregulation (CA) status on outcome in both AIS and ICH.^20^ Specifically, meta-analyses have demonstrated that dynamic CA in both affected and unaffected hemispheres is significantly impaired in all stroke types when compared to control subjects, though remains intact in the unaffected hemisphere in large territory stroke. Furthermore, specifically in ICH, the affected hemisphere has lower mean cerebral blood flow velocity and impaired CA, which may have implications for those being treated with intensive BP lowering.^21^ Currently, we acknowledge that a complex interplay exists between autoregulatory impairment, treatments including blood pressure lowering and clinical outcome.^22^ However, limited studies to date have examined strategies including individualised thresholds of autoregulation and outcome or intervening specifically to improve autoregulatory status.^20^ Ultimately, further research is needed to robustly test autoregulatory focussed treatment strategies, effects of BP lowering on autoregulatory status and continuous ward based autoregulatory monitoring.^23^

## Integrating mechanistic insights with interventional trials

In order to robustly test blood flow response and autoregulatory function during BP lowering in acute stroke, we need to demonstrate clear relationships. Recent data has suggested BP levels in older people with hypertension have limited influence on cerebral blood flow (assessed using arterial spin labelling) in the short term.^24^ However, can we expect there to be greater disturbance of this relationship where greater cerebral tissue disruption exists (acute ischaemic or haemorrhagic stroke)? There is an argument for differentiating chronic hypertensives from those with an immediate hypertensive response in hyperacute ischaemic or haemorrhagic stroke as autoregulatory function particularly may differ significantly at presentation.^21^,^25^ This is particularly relevant given advanced imaging techniques and a greater emphasis now on reperfusion risks and complications, many of which are precipitated and worsened by uncontrolled hypertension.^26^ Near-infrared spectroscopy-derived tissue oxygenation to assess fixed compared with autoregulation-orientated BP thresholds post mechanical thrombectomy for ischemic stroke has been deemed feasible.^27^ Crucial lessons from this study include the demonstration of infarct progression where the lower limit of autoregulation is breached – demonstrating the necessity for maintenance of cerebral perfusion to sustain ischaemic brain tissue.^27^ We need dynamic autoregulatory function measurement post stroke and consideration for a personalised “optimum” for which all interventions post stroke including blood pressure therapies are tailored, particularly as in some instances the data may be telling us that less is perhaps more?

## Future work

In acute ischaemic stroke specifically, there remains a paucity of randomised data on blood pressure lowering in those with the greatest severity. Although pre-specified sub-group analyses of trial data have shown increased mortality during intensive BP lowering in severe acute ischaemic stroke (large atheromatous disease or National Institutes of Health Stroke Scale >10) despite lowered risk of ICH, further randomised data are needed.^28^ ENCHANTED2 (*ClinicalTrials.gov* NCT04140110) is an international, multicentre, prospective, randomised trial assessing different intensities of blood pressure lowering in those undergoing mechanical thrombectomy. Specifically, comparing an intervention group with SBP <120 mmHg within 1 hour against a control group maintaining SBP 140 mmHg–180–mmHg, with both groups maintained at targets for at least 72 hours. Crucially, despite prospective non-randomised studies of BP lowering post mechanical thrombectomy demonstrating positive benefits in those who successfully recanalise, there still remains uncertainty.^29^,^30^ In acute haemorrhagic stroke, further work is necessary to understand within the ultra-acute and acute period, during which haematoma expansion occurs, whether intervention strategies including BP lowering are safe and whether specific agents and delivery methods are key to better outcomes.

## Conclusion

Large clinical trials have tested early BP reduction (<48 hours) in the management of acute ischaemic and haemorrhagic stroke. These trials inform current guidelines and overall demonstrate acceptable safety of BP lowering in these stroke sub-types. These trials have informed areas requiring further consideration in the future, including: specific timing of lowering, choice of agent and ultimately highlighting the necessity for better phenotyping of those who appear to be benefiting from this simple but effective intervention. Integrating mechanistic considerations with clinical interventional trials represents the next phase of research interest as development of personalised interventions necessitate careful consideration of blood flow parameters.
